# Amy63, a novel type of marine bacterial multifunctional enzyme possessing amylase, agarase and carrageenase activities

**DOI:** 10.1038/srep18726

**Published:** 2016-01-04

**Authors:** Ge Liu, Shimei Wu, Weihua Jin, Chaomin Sun

**Affiliations:** 1 Key Laboratory of Experimental Marine Biology, Institute of Oceanology, Chinese Academy of Sciences, Qingdao 266071, China; 2 University of Chinese Academy of Sciences, Beijing, 100049, China; 3 Laboratory for Marine Biology and Biotechnology, Qingdao National Laboratory for Marine Science and Technology, Qingdao, 266071, China; 4 Key Laboratory of Biobased Materials, Qingdao Institute of Bioenergy and Bioprocess Technology, Chinese Academy of Sciences, Qingdao, 266101, China

## Abstract

A multifunctional enzyme is one that performs multiple physiological functions, thus benefiting the organism. Characterization of multifunctional enzymes is important for researchers to understand how organisms adapt to different environmental challenges. In the present study, we report the discovery of a novel multifunctional enzyme Amy63 produced by marine bacterium *Vibrio alginolyticus* 63. Remarkably, Amy63 possesses amylase, agarase and carrageenase activities. Amy63 is a substrate promiscuous α-amylase, with the substrate priority order of starch, carrageenan and agar. Amy63 maintains considerable amylase, carrageenase and agarase activities and stabilities at wide temperature and pH ranges, and optimum activities are detected at temperature of 60 °C and pH of 6.0, respectively. Moreover, the heteroexpression of Amy63 dramatically enhances the ability of *E. coli* to degrade starch, carrageenan and agar. Motif searching shows three continuous glycosyl hydrolase 70 (GH70) family homologs existed in Amy63 encoding sequence. Combining serial deletions and phylogenetic analysis of Amy63, the GH70 homologs are proposed as the determinants of enzyme promiscuity. Notably, such enzymes exist in all kingdoms of life, thus providing an expanded perspective on studies of multifunctional enzymes. To our knowledge, this is the first report of an amylase having additional agarase and carrageenase activities.

Multifunctional enzymes (MFEs) are enzymes that are considered to perform multiple independent functions, and are often also moonlighting or promiscuous enzymes^1^,^2^,^3^,^4^,^5^. Moonlighting enzymes perform multiple autonomous and unrelated functions that are not due to gene fusions, multiple RNA splice variants, or pleiotropic effects^3^. Promiscuous enzymes can catalyze fortuitous side reactions in addition to their main or native reactions, although these secondary reactions are usually slow relative to the main activity and are under neutral selection^6^. In particular, substrate promiscuous enzymes are enzymes with relaxed or broad substrate specificity which can switch catalytic activities under different reaction conditions^2^. MFEs are beneficial to living organisms since they expand the biological functions of an organism without the burden of an expanding genome^7^. Moreover, multifunctionality can provide a switch point in biochemical or signaling pathways to enable organisms to better adapt to their environment^8^.

Since the first multifunctional enzyme, crystallins, was reported by Piatigorsky and Wistow^9^, discovery of novel MFEs has increased^3^. Notably, MFEs are described in diverse species including animals, plants, yeasts and prokaryotes^2^, suggesting that MFEs broadly exist in all kingdoms of life. Moreover, a growing number of MFEs have been found to play key roles in disease^3^. Therefore, recent novel methods combining biochemistry with bioinformatics have been developed to find novel MFEs^2^.

α-amylase represents the most intensively studied amylolytic enzyme, which degrades starch substrates and is applied widely in various branches of the food, pharmaceutical, and chemical industries^10^,^11^,^12^. Most α-amylases (EC 3.2.1.1) belong to the family 13 of glycoside hydrolases (GH13), which forms the GH-H clan together with the families GH70 and GH77. However, the latter contain no α-amylases^10^. The polyspecific family GH13 covering more than 30 different amylolytic and related enzyme specificities with nearly 16,000 sequences ranks among the largest of the GH families^13^. Multifunctional amylases such as maltogenic amylases (EC 3.2.1.133) constitute a special subfamily in the framework of the α-amylase family or GH-13 family because they have some unique catalytic and/or structural characteristics compared with other enzymes of the α-amylase family or GH-13 family^14^. Multifunctional amylases exhibit both transglycosylation and hydrolysis activities on various glucan substrates, leading to the production of isomaltooligosaccharides and maltooligosaccharides as well as glucose^5^. For example, the oligosaccharide-producing multifunctional amylase (OPMA) has strong α-1, 6-transglycosylation activity in addition to its α-1, 4-hydrolytic activity on starch and some oligosaccharides^5^,^14^.

In this study, we report a novel multifunctional α-amylase Amy63, isolated from *Vibrio alginolyticus* 63, which possesses agarase and carrageenase activities in addition to its main amylase activity towards soluble starch, amylose, amylopectin and glycogen. The heteroexpression of Amy63 dramatically enhances the degradation abilities of *E. coli* towards starch, carrageenan and agar. Serial deletions together with sequence analysis of Amy63, lead us to propose that the GH70 homologs determine multifunctionality. Phylogenetic analysis of Amy63 indicates that this kind of multifunctional amylases is widely spread in the all kingdoms of life, which profoundly broaden our knowledge about the α-amylase.

## Results

### The discovery of the multifunctional enzyme Amy63

**Figure 1:**
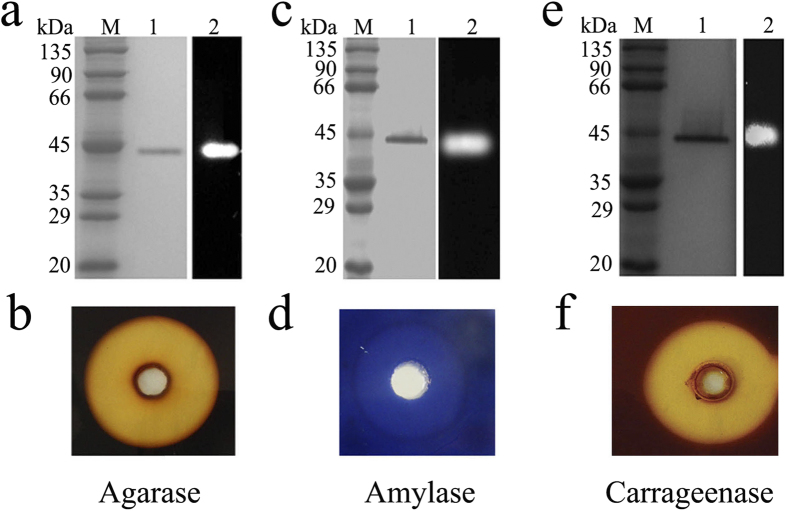
Zymogram analyses and plate-based activity assays of agarase, amylase and carrageenase activities of native Amy63. Zymogram analyses of agarase (**a**), amylase (**c**) and carrageenase (**e**) activities of native Amy63; Plate-based activity assays of agarase (**b**), amylase (**d**) and carrageenase (**f**) properties of native Amy63. Lane M, molecular mass markers; Lane 1, purified native Amy63; Lane 2, zymogram of purified native Amy63.

The initial purpose of this study was to identify robust marine bacterial agarases. By screening crude extracts, we discovered that the agarase produced by marine bacterium strain 63 exhibited the highest activity and stability in various temperatures and pHs, even in presence of surfactants or chelating agents. Therefore, we purified the native agarase, hereafter named Amy63, from the producing strain and confirmed it was functional by zymogram analysis (Fig. 1a) and a plate-based activity assay (Fig. 1b). In view of the high homology (99% identity) with marine bacterium *Vibrio alginolyticus* by the 16S rRNA gene sequencing (Accession no. KT224384), the Amy63-producing strain was designated as *Vibrio alginolyticus* 63.

To determine the protein sequence of Amy63, MALDI-TOF⁄TOF mass spectrometry was performed on the purified native protein. Surprisingly, the sequencing results from the mass spectrometry showed that Amy63 has high homology with the cytoplasmic α-amylase of *Vibrio alginolyticus* (Accession no. gi|491538798). To check whether Amy63 had amylase activity, zymogram analysis and plate-based activity assays were performed. Remarkably, the results showed that purified Amy63 had strong amylase activity which effectively degraded starch in a PAGE gel (Fig. 1c) and an agar plate (Fig. 1d). Moreover, Amy63 was found to also possess carrageenase activity (Fig. 1e,f). Taken together, Amy63 is a multifunctional enzyme possessing amylase, agarase and carrageenase activities.

### Multifunctionality verification with the recombinant Amy63 in *E. coli*

Purified native Amy63 displays multifunctionality in our assays; however, we wanted to ensure that this was not due to potential co-purification of amylase or carrageenase. The engineered *E. coli* BL21(DE3) is an ideal system for testing this because it provides a background which does not express any agarases, amylases or carrageenases. To heterologously express Amy63 in *E. coli* BL21(DE3), we first cloned the coding sequence of Amy63 (Accession no. KT224383) based on the mass spectrometry results (Supplementary Figure S1) as described in the Methods. The sequence showed that Amy63 was encoded by 507 amino acids, with a calculated molecular weight of 58.5 kDa, which was in agreement with the mass estimated by SDS-PAGE. Thereafter, Amy63 was overexpressed in *E. coli* and purified through nickel, anion exchange, and gel filtration columns. The purified recombinant Amy63 appeared as a single band in PAGE gel and a clear zone formed around the protein band in agar, starch and carrageenan containing gel sheets (Supplementary Figure S2a, S2c, S2e). These results were further verified by plate-based activity assays (Supplementary Figure S2b, S2d, S2f). Together, we have confirmed that recombinant Amy63 is a multifunctional enzyme with amylase, agarase and carrageenase activities, which is consistent with the characteristics of native Amy63.

### Amy63 is a typical α-amylase with promiscuous agarase and carrageenase activities

**Figure 2:**
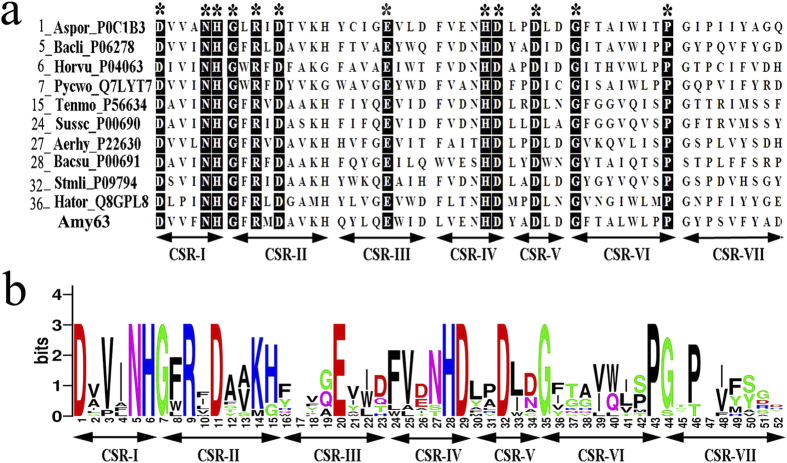
Sequence analyses of Amy63. (**a**) Amino acid sequence alignment of seven conserved sequence regions (CSRs) in Amy63 and other GH13 α-amylases representing the individual α-amylase subfamilies. Conserved residues in all sequences are indicated by asterisks. The conserved domains are numbered I–VII at the bottom of the figure. The name of an enzyme used for sequence analysis is composed of the GH13 subfamily number followed by the abbreviation of the source (organism) and the UniProt accession number. The organisms are abbreviated as follows: Aspor, *Aspergillus oryzae*; Bacli, *Bacillus licheniformis*; Horvu, *Hordeum vulgare*; Pycwo, *Pyrococcus woesei*; Tenmo, *Tenebrio molitor*; Sussc, *Sus scrofa* (pancreas); Aerhy, *Aeromonas hydrophila*; Bacsu, *Bacillus subtilis*; Stmli, *Streptomyces limosus*; Hator, *Halothermothrix orenii*. (**b**) Sequence logo for the seven CSRs of the GH13 subfamilies α-amylases. The size of a single letter amino acid code in the sequence logo represents the occurrence of a particular amino acid at a particular position.

We next further characterized the multifunctional activities of Amy63. First, we carefully analyzed the encoding sequence of Amy63. By comparing the sequence to ten representative α-amylases from GH13 subfamilies 1, 5, 6, 7, 15, 24, 27, 28, 36, 37, seven conserved sequence regions (CSRs) typical for α-amylase were identified in Amy63 (Fig. 2a) and a WebLogo covering seven CSRs was created (Fig. 2b). However, Amy63 did not have homology to any agarase or carrageenase in NCBI protein database. Therefore, we conclude that Amy63 is a typical GH13 family α-amylase.

**Figure 3:**
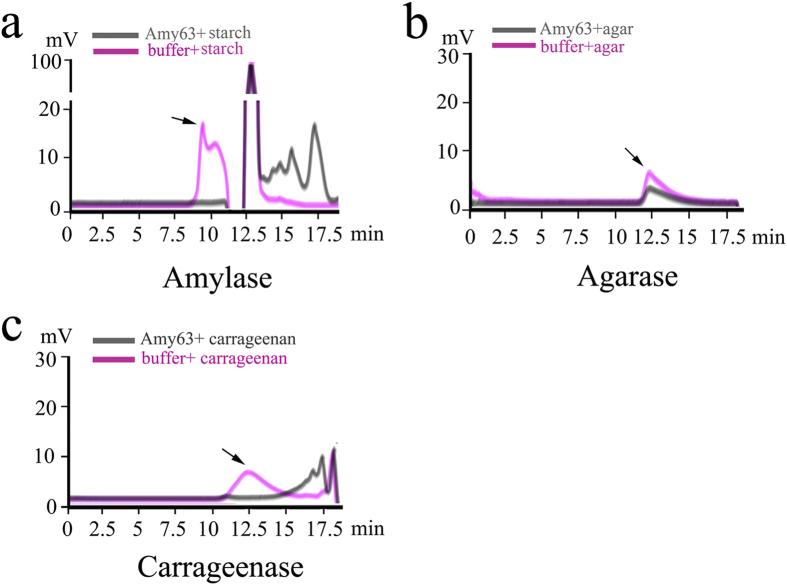
Starch (**a**), agar (**b**) and carrageenan (**c**) degradation analyses of Amy63 measured by gel permeation chromatography. The arrows indicate the peaks of starch, agar and carrageenan.

To measure the activities of Amy63 on its substrates, the changes in the average molecular weights of starch, agar and carrageenan in a hydrolysis reaction mixture were measured using gel permeation chromatography (GPC) as described by Suzuki *et al.*^15^. The average molecular weights of soluble starch and carrageenan decreased significantly after the addition of Amy63, which were consistent with the results of zymogram and plate-based activity assays (Fig. 3a,c). However, for agar, the average molecular weight only dropped slightly after incubation with Amy63 (Fig. 3b), suggesting this is likely a promiscuous activity. Notably, this is in agreement with studies of other promiscuous enzymes. For example, the *kcat/KM* values of the promiscuous sugar kinase *YajF* is approximately 10^2^ M^−1^s^−1^ and thus ~10^4^ lower than that of the primary *E. coli* glucokinase (*Glk*)^16^. Thus, combining the sequence analysis, zymogram and plate-based activity assays and GPC results, we conclude that Amy63 is a typical α-amylase possessing promiscuous agarase and carrageenase activities, and the priority order of substrate usage for Amy63 is soluble starch, carrageenan and agar.

### Identification of the substrate specificity and maltohexaose degradation capability of Amy63

**Figure 4:**
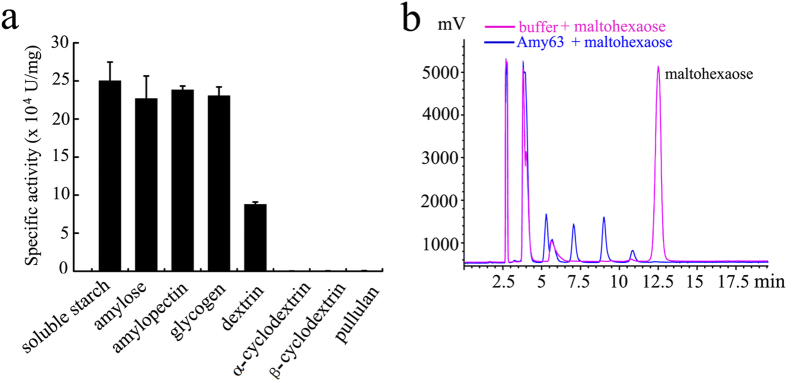
Substrates specificity and maltohexaose degradation capability analyses of Amy63. (**a**) Analyses of the substrate specificity of Amy63 against different glucan substrates using DNS methods. Values represent the mean ± SD of six cultures (n = 3) performed in triplicate. (**b**) Maltohexaose degradation assay of Amy63 analyzed by hydrophilic interaction chromatography.

Because the amylase activity is the primary function of Amy63, we next sought to identify the substrate specificity of amylase activity of Amy63. We tested the enzyme against different glucose polymers and found that Amy63 has wide substrate specificity, with soluble starch being the best substrate (Fig. 4a). Amy63 exhibited high activity towards soluble starch, amylose, amylopectin and glycogen but had extremely low activity on α-cyclodextrin, β-cyclodextrin and pullulan. This suggests that Amy63 is an endolytic amylase, which is analogous to the α-amylase from *Marinobacter sp.* EMB8^17^. Next, we checked whether Amy63 could degrade maltohexaose to form lower molecular weight oligosaccharides. The hydrophilic interaction chromatography results showed that purified Amy63 produced oligosaccharides with a degree of polymerization of 1 to 5 from maltohexaose after 24 h of hydrolysis (Fig. 4b). This capability of Amy63 further indicated that it possessed an endo-type mechanism of amylase action.

### Characterization of the multifunctional enzyme Amy63

**Figure 5:**
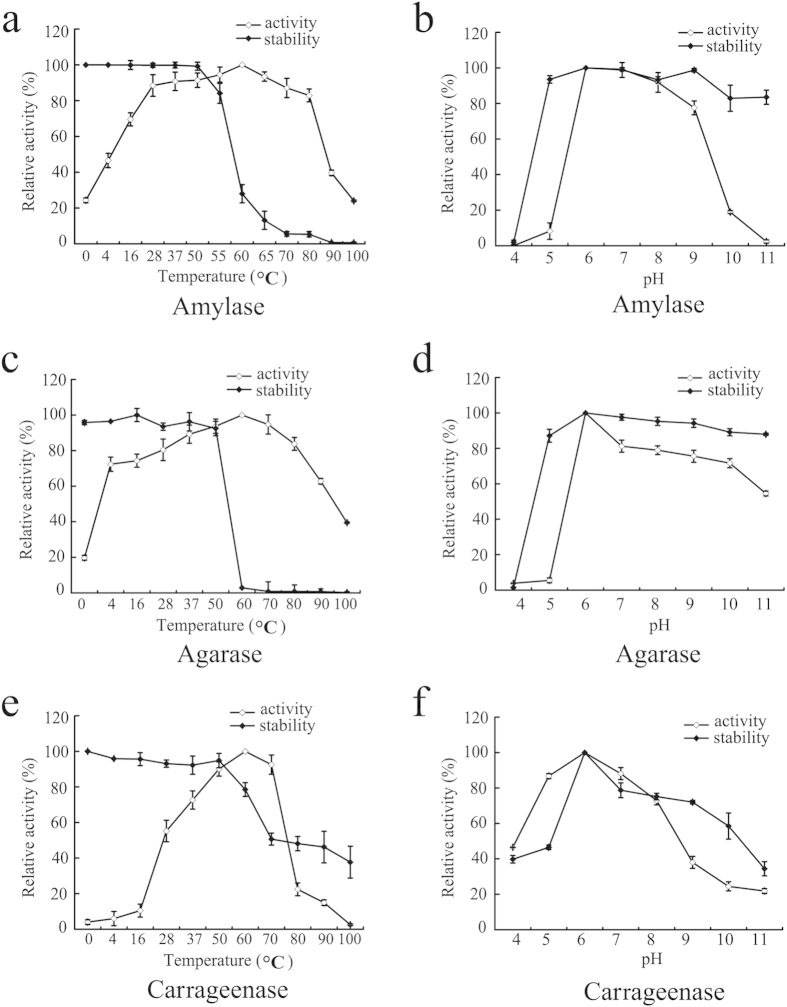
Effects of temperature and pH on amylase activity (**a**) and stability (**b**), agarase activity (**c**) and stability (**d**) and carrageenase activity (**e**) and stability (**f**) of Amy63. Values represent the mean ± SD of six cultures (n = 3) performed in triplicate.

Because temperature and pH are decisive parameters for enzyme activity^18^, we characterized the effects of altering temperature and pH on the amylase, agarase and carrageenase activities and stability of Amy63 by using DNS methods, as described in the Methods. The optimal temperature for Amy63 amylase activity was 60 °C, and this activity was stable up to 55 °C (Fig. 5a). The optimum pH for Amy63 amylase activity was 6.0, which is more acidic than the optimal pH of any other reported α-amylase^18^,^19^. This activity remained stable in the pH range from 5.0 to 11.0 (Fig. 5b). The effects of temperature and pH on agarase or carrageenase activity and stability of Amy63 were also checked. Similar to the amylase properties, Amy63 exhibited maximum agarase and carrageenase activities at 60 °C (Fig. 5c,e) and pH 6.0 (Fig. 5d,f).

**Figure 6:**
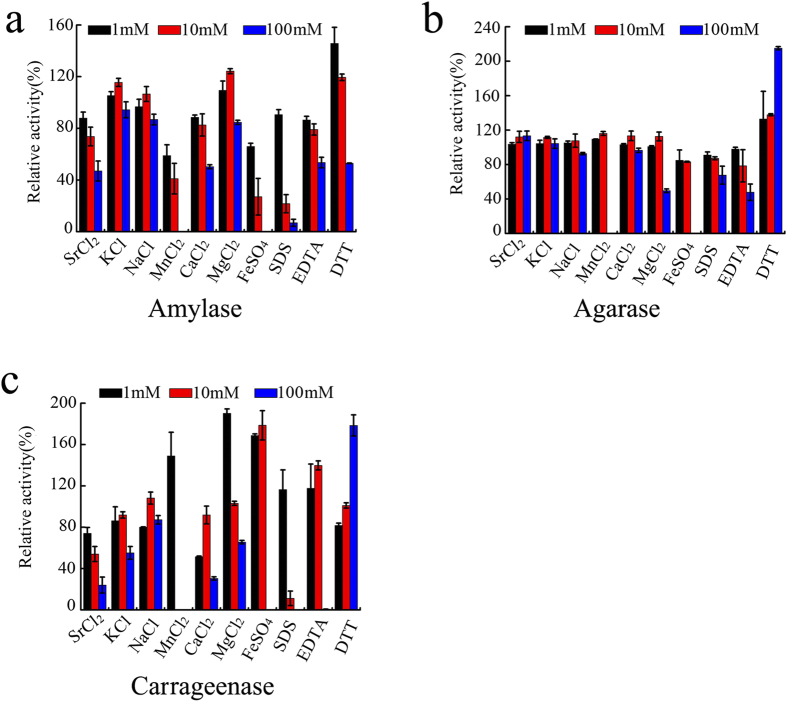
Effects of metal ions, surfactant and chelating agent on amylase (**a**), agarase (**b**) and carrageenase activities (**c**) of Amy63. Values represent the mean ± SD of six cultures (n = 3) performed in triplicate.

The effects of metal ions and other reagents on amylase, agarase and carrageenase activities of Amy63 were also investigated (Fig. 6). The activities were stable in conditions with 100 mM macroelements found in seawater, such as Na^+^ and K^+^ (Fig. 6a–c). Amy63 maintained its full agarase activities in 100 mM Sr^2+^ and Ca^2+^ (Figure 6b), but not its amylase (Fig. 6a) and carrageenase (Fig. 6c) activities. Ten mM Mn^2+^ could activate the agarase activity (Fig. 6b) but weakened its amylase (Fig. 6a) and carrageenase (Fig. 6c) activities, while increasing to 100 mM Mn^2+^ completely abolished all three enzymes activities (Fig. 6). Amy63 maintained all three activities in the presence of 10 mM Mg^2+^ but lost ~50% activity at 100 mM Mg^2+^ (Fig. 6). Ten mM Fe^2+^ greatly activated the carrageenase activity (Fig. 6c) but decreased the amylase activity (Fig. 6a). Low concentration (1 mM) of detergent did not affect the enzymatic activities of Amy63; however, 100 mM of SDS dramatically decreased the amylase and carrageenase activities (Fig. 6a,c). High concentration (100 mM) of the chelating agent EDTA abolished all carageenase activity (Fig. 6c) but retained considerable amylase and agarase activities (Fig. 6a, 6b). Surprisingly, the amylase activity of Amy63 decreased with increasing concentrations of DTT from 1 to 100 mM (Fig. 6a), while in contrast the agarase (Fig. 6b) and carrageenase (Fig. 6c) activities were activated. Collectively, the amylase, agarase and carrageenase activities of Amy63 respond differently to each condition, indicating that Amy63 might prioritize each enzymatic function for different physiological responses.

### Analysis of the physiological functions of Amy63

**Figure 7:**
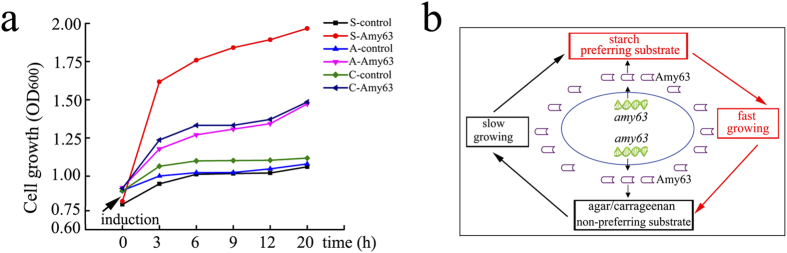
Physiological function assays of Amy63. (**a**) Growth rates of *E. coli* BL21(DE3) cells with or without expressing Amy63 in basic medium supplemented with starch, agar or carageenan as the sole-carbon source. The arrow indicates the time point of induction. S-control indicates cells without expressing Amy63 growing in basic medium supplemented with starch; S-Amy63 indicates cells expressing Amy63 growing in basic medium supplemented with starch; A-control indicates cells without expressing Amy63 growing in basic medium supplemented with agar; A-Amy63 indicates cells expressing Amy63 growing in basic medium supplemented with agar; C-control indicates cells without expressing Amy63 growing in basic medium supplemented with carrageenan; C-Amy63 indicates cells without expressing Amy63 growing in basic medium supplemented with carrageenan. (**b**) Proposed physiological function model of Amy63. With the preferring substrate starch, the Amy63 producing bacteria could get enough carbon source to have high growth rate. However, in some harsh environment without enough starch, the bacteria have to take advantage of the multifunctionality of Amy63 to utilize non-preferring substrates such as agar and carrageenan as the carbon source for surviving. Once the bacteria get over the hard time and have enough starch around, the amylase activity of Amy63 would function again and make the bacteria for another round fast growing.

We next asked what physiological roles Amy63 contributes to for the producing bacterium. We performed these functional assays in the *E. coli* BL21(DE3) system because it has no any amylase, agarase and carrageenase activities. In view of Amy63’s multifunctionality, we hypothesized that the expression of Amy63 could enhance the degradation abilities of *E. coli* cells towards starch, agar or carrageenan, thus increasing the carbon source. If this were the case, then we would expect the host cells to grow faster in the basic medium supplemented with starch, agar or carrageenan as the sole-carbon source upon expression of Amy63. As expected, the growth rates of *E. coli* cells in the different mediums were very similar before the expression of Amy63; however, the growth rates changed dramatically once Amy63 was expressed (Fig. 7a). Notably, in the basic medium supplemented with starch, twenty hours after the expression of Amy63, the OD600 value of cells containing Amy63 was almost two times to that of cells not expressing Amy63. Similarly, in the basic medium supplemented with agar or carrageenan, the *E. coli* cells grew faster in presence of Amy63 (Fig. 7a), and the OD600 value of cells was around 1.5 times greater than in cells without Amy63. When comparing the growth rates of cells containing Amy63 in the basic media supplemented with starch, carrageenan and agar, we note that the utilizing abilities of host to starch (Fig. 7a), carrageenan and agar are consistent with those of Amy63 analyzed *in vitro* (Fig. 3). Collectively, the expression of Amy63 renders the host capable of degrading starch, agar and carrageenan to become the carbon source for growth.

### Functional domains determination and evolution analyses of multifunctional enzyme Amy63

**Figure 8:**
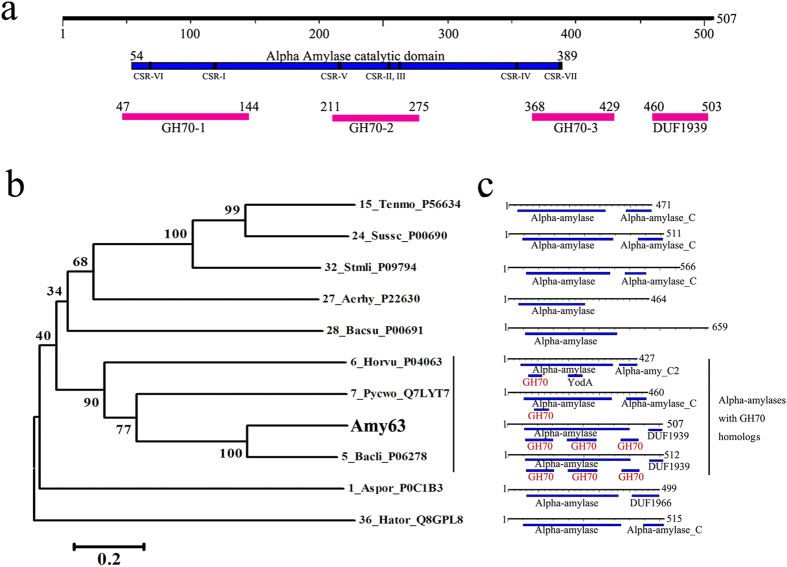
Phylogenetic analysis of Amy63 with other typical GH13 subfamily α-amylases with or without GH70 homologs. (**a**) The domain organization of Amy63 analyzed with MOTIF Search program. The numbers represent the amino acid numbers of corresponding protein and domains. (**b**) Unrooted phylogenetic tree of Amy63 with other typical GH13 subfamily α-amylases with or without GH70 homologs. The α-amylases used for the phylogenetic analysis are the same to those used for the CSRs alignment in Figure 2. Alignments and dendrogram construction were carried out with MEGA version 6.0, using the neighbor joining method. Bootstrap values (expressed as percentages) are given at the branching points. The bar corresponds to a genetic distance of 0.2 substitution per position (20% amino acid sequence difference). (**c**) The corresponding domain organization of GH13 subfamily α-amylases used in panel b. There is a one-to-one relationship between panel (**b,c**). All the domain organizations of GH13 subfamily α-amylases were analyzed with MOTIF Search program.

To understand Amy63’s multifunctionality, we analyzed the sequence of Amy63 with Motif Search program (http://www.genome.jp/tools/motif/), and three conserved GH70 homologs and a DUF1939 domain were identified in the polypeptide chain of Amy63 (Fig. 8a). Using twenty-one typical α-amylases possessing three conserved GH70 homologs and DUF1939 domain, the WebLogo covering three conserved GH70 homologs and DUF1939 domain was created (Supplemental Figure S3b). The WebLogo results showed that there were many absolutely conserved amino acids within the three GH70 homologs. It is noteworthy that the twenty-one typical α-amylases using for WebLogo analysis were from bacteria, fungi and archaea.

In order to disclose the possible functional domain responsible for the multifunctional property of Amy63, three GH70 homologs and DUF1939 domain were thus deleted one by one and then the enzymatic activities against starch, agar and carrageen were measured, respectively. Surprisingly, Amy63 had similar agarase, amylase and carrageenase activities even when only one GH70 homolog was kept, when compared to when two or three GH70 homologs kept (Supplemental Figure S3a). Therefore, we proposed that the multifunctional property of Amy63 was not determined by a specific domain and its promiscuity was due to the gene fusion of GH70 homolog (see Discussion).

To determine the evolutionary status of Amy63, a phylogenetic tree was constructed using Amy63 and ten typical α-amylases from GH13 subfamilies 1, 5, 6, 7, 15, 24, 27, 28, 36, 37 (Fig. 8b). The results revealed that α-amylase with GH70 homologs formed a tight polygenetic cluster in the tree and Amy63 was most closely related to *Bacillus licheniformis* α-amylase (GenBank Accession No. P06278), which has a very similar GH70 homologs distribution pattern with Amy63 (Fig. 8c).

Moreover, we checked all the α-amylases published in the website of http://www.cazy.org. Notably, motif searching results with MOTIF Search program showed that the GH70 homolog(s) containing α-amylases ubiquitously exist in all kingdoms of life (Supplemental Figure S4), which indicates that undiscovered multifunctional α-amylases may exist in many other organisms. Moreover, α-amylases with three continuous GH70 homologs cluster together with Amy63 in the phylogenetic tree, regardless of the life domain of the α-amylase (Supplemental Figure S4).

## Discussion

Traditionally, enzymes are highlighted for having specific activities, while enzyme cross-reactivity or promiscuity has been largely ignored^6^,^20^. However, recent evidence suggests that enyzmes with promiscuity can have important functions^6^. Furthermore, research into promiscuity leads to interesting insights, in particular by studying the catalytic mechanisms of promiscuity and the evolvability of promiscuous functions^6^.

In this study, we discovered and characterized a novel marine bacterium multifunctional enzyme, Amy63, and showed that it was a typical α-amylase with promiscuous agarase and carrageenase activities. The evidence for Amy63 promiscuity are based on the following observations: (i) Both native and recombinant Amy63 have strong hydrolytic activities on agar and carrageenan in addition to starch by zymogram anyalyses and plate-based activity assays; (ii) Amy63 shares seven typical conserved sequence regions (CSRs) of α-amylase and phylogenetically clusters with the typical GH13 family α-amylases; (iii) Gel permeation chromatography results further confirm Amy63 could degrade starch, agar and carrageenan. Moreover, gel permeation chromatography results indicate that starch is the best substrate of Amy63, and the degradation capabilities of agar and carrageenan by Amy63 are weaker than that of starch. Therefore, we conclude that Amy63 is a substrate promiscuous enzyme. The priority order for substrates of Amy63 is starch, carrageenan and agar. To our knowledge, there are no other enzymes with such activities reported thus far.

By characterizing Amy63, we found that the optimal pH and temperature of Amy63 is around 6.0 and 60 °C, respectively. Moreover, the enzymatic activity is stable within the pH range from 5.0 to 11.0 and the temperature range between 0 and 70 °C. In addition to starch, hydrolysis product analysis showed that carrageenan and agar could be degraded into glycans with a lower degree of polymerization by Amy63. This suggests Amy63 could have potential applications in many industrial processes such as starch or other complex substrate saccharification containing agar or carrageenan in sugar and textile industries^21^.

The effects of metal ions and other reagents on activity of Amy63 were also checked. It is noteworthy that DTT dramatically enhanced Amy63’s activity, especially for its agarase and carrageenase activities, which is similar to ScAmy43^22^. DTT could cause the better accessibility of the substrate to the catalytic site after disruption of the intermolecular disulfide bridge^23^. However, no cysteine residues have been found in the amino acid sequences of Amy63 indicating that DTT could affect Amy63 function by forming stable complexes with metal ions^24^ or inducing conformational changes^25^. For Amy63, its amylase, agarase or carrageenase activities had different responses to the same reagent, indicating that this multifunctional enzyme might exhibit each enzyme activity through different mechanisms. Indeed, promiscuous enzymatic functions can utilize different active-site conformers, or differ altogether from the mechanism by which an enzyme performs its native function^6^.

The gene sharing model, where a gene with a given function is recruited for a different, moonlighting function without any changes in the coding region^9^, is often used to explain why an enzyme maintains promiscuity. Sequence analyses indicated that Amy63 contains three continuous GH70 homologs. The GH70 (http://www.cazy.org) family enzymes displayed a variety of catalytic activities, including dextransucrase (EC 2.4.1.5), alternansucrase (EC 2.4.1.140), reuteransucrase (EC 2.4.1.-), α-4,6-glucanotransferase (EC 2.4.1.-) and α-1,2-branched dextransucrase (EC 2.4.1.-). The enzymes from glycoside hydrolase families GH13, GH70, and GH77 together form clan GH-H. GH13 family amylosucrase enzymes have the GH13 type of domain architecture and the GH70 type of glucansucrase activity^26^. GH70 family enzyme GTFB has GH70 domain architecture and the GH13 amylolytic activity^27^. Therefore, gene and function fusions between GH13 and GH70 families exist in nature. Because of our results from the GH70 homologs deletion experiment, we propose that GH70 homologs might play an important role in the multifunctionality of Amy63. However, the exact relationship between GH70 homologs and the multifunctionality of Amy63 need to be elucidated further in the future.

MFEs are able to employ alternative approaches to coordinate multiple activities and regulate their own expression which could benefit the living systems by providing competitive survival capabilities^28^. The existence of MFEs were demonstrated to be an evolutionary advantage as part of a clever strategy for generating complexity from existing proteins without expansion of the genome^29^. Multifunctionality enables an enzyme to act as a switch point in biochemical or signaling pathways, thus allowing a cell to rapidly respond to changes in surrounding environment^8^. Furthermore, evolution of the active sites of existing enzymes that promiscuously bind the substrates can allow new enzymatic capabilities to be generated^6^. The marine bacterium strain producing Amy63 was isolated from algae in South China sea. Because algae is rich in many kinds of starches^30^, it is possible that bacteria living together with algae evolved to use starch as the main carbon source. Accordingly, these bacteria likely rely most on starch degradation enzymes like amylase. However, during times like in the winter, the bacteria must survive in a harsh environment without enough starch. Thus, we hypothesize that these marine bacteria have evolved their existing amylase to allow usage of other carbohydrates like agar and carrageenan as the carbon source (Fig. 7b).

Multifunctional α-amylases, like Amy63, may generally exist in the biological world, since GH70 homolog containing α-amylases are ubiquitous in all kingdoms of life. For Amy63, it will be important to investigate the detailed mechanisms of catalytic promiscuity and the evolvability of promiscuous functions in the future.

## Methods

### Isolation and identification of marine bacterium strain 63

Marine bacterial strains were isolated from South China Sea and screened for their abilities to hydrolyze agar by streaking strain in the 2216E agar plate containing 5 g/l tryptone, 1 g/l yeast extract, and 20 g/l agar, one liter filtered seawater, pH adjusted to 7.4–7.6. The plate was incubated at 28 °C for 48 h and stained with Lugol’s iodine solution (5% I_2_ and 10% KI in distilled water). Colonies with a clear zone formed by the hydrolysis of agar were evaluated as agarase producers. Marine bacterium strain 63 was isolated from algae of South China sea, inoculated in marine broth 2216E, and grown at 28 °C. Genomic DNA was extracted from the isolate, and PCR (polymerase chain reaction)^31^ was performed to amplify the 16S rRNA gene sequence with universal primers 27F and 1541R. And the 16S rRNA gene sequence was analyzed by using the BLAST programs (http://blast.ncbi.nlm.nih.gov/Blast.cgi) to determine the phylogenetic position of the bacterium strain 63.

### Production and purification of native Amy63

Amy63 was purified as described previously^32^,^33^ with minor modification. Briefly, marine bacterium strain 63 was propagated in marine broth 2216E containing 0.1% agar for 48 h at 28 °C. The culture broth was obtained by centrifugation at 8,000 rpm for 20 min at 4 °C and was adjusted to 80% saturation with ammonium sulfate. After precipitation overnight, the mixture was centrifuged (10,000 rpm g for 20 min) and the collected precipitate was dissolved in 10 mM Tris-HCl buffer (pH 8.0) and dialyzed against buffer A (100 mM NaCl in 10 mM Tris-HCl, pH 8.0) overnight. The dialyzed solution was applied to a HiTrap^TM^ Q HP column (GE Healthcare) pre-equilibrated with buffer A. Bound proteins were eluted with 150 ml linear gradient of 0.1–1.0 M of NaCl in 10 mM Tris-HCl (pH 8.0) at a flow rate of 5 ml/min. Active fractions were collected and concentrated by ultrafiltration (MWCO 10 kDa, Millipore), and loaded onto a Hiload^TM^ 16/600 superdex^TM^ 200 column (GE Healthcare) pre-equilibrated with 10 mM Tris-HCl (pH 8.0) containing 0.15 M NaCl. Bound proteins were eluted with the equivalent buffer at a flow rate of 1 ml/min (2 ml per tube). Active fractions were collected for further study. All purifications were carried out at 4 °C using an AKTA purifier system (Amersham Biosciences, Piscataway, NJ, USA).

### Plate-based activity assays and zymogram analyses of Amy63

For plate-based activity assays of Amy63, the same amount of purified Amy63 was put in the center hole of plates containing 2% agar, 5% carrageenan, or 1% soluble starch with 1.5% agar plus 0.01% Trypan Blue in 20 mM Tris–HCl buffer (pH 8.0), respectively. The plates were kept in 50 °C for 2 h. Plates containing agar and carrageenan for checking agarase and carrageenase activities were stained with Lugol’s iodine solution and then visualized the clear zone around the hole. Plates containing soluble starch and Trypan Blue were checked directly for the amylase activity by visualization of clear zone around the hole.

For zymogram analyses of Amy63, the same amount of purified Amy63 was performed with SDS-PAGE on a 10% gel as described by Laemmli^34^. After electrophoresis, the gel was soaked in 20 mM Tris–HCl buffer (pH 8.0) for a total period of 30 min to remove the SDS and the soaking buffer was changed three times during this period. Then the gel was overlaid on a sheet containing 2% agar, 5% carrageenan, or 1% soluble starch with 1.5% agar and 0.01% Trypan Blue in 20 mM Tris–HCl buffer (pH 8.0) for 2 h at 50 °C. Finally, those sheets were stained by Lugol’s iodine solution. Agarase, carrageenase, or amylase activity was visualized as a clear zone on the brown or blue background, respectively.

### Protein MALDI-TOF⁄TOF Mass Spectrometry

The procedure of MALDI-TOF⁄TOF mass spectrometry was carried out as described previously^35^. Briefly, the CBB-stained protein was manually excised from the gel and then digested *in situ* with trypsin using established methods^36^. After digestion overnight at 37 °C, 5% formic acid (v/v) was added to stop the reaction and the peptides were extracted from the mixture. Then the desalted samples were spotted by a MALDI-TOF ⁄TOF tandem mass spectrometer, and the primary mass data were acquired.

### Gene cloning and sequence analysis of encoding sequence of Amy63

Based on the first peptide RVDWNNR and last peptide QHSYLDHWDVIGWTR identified from Mass spectrometry, a pair of degenerate primers P1F and P1R were designed to amplify the corresponding DNA. The amplified PCR products were sequenced and blasted the homology in NCBI. The sequence alignments result showed that it had high homology (99%) with cytoplasmic α-amylase of *Vibrio alginolyticus* (Accession no. gi|491538798). Therefore, a pair of degenerate primers P2F and P2R based on the gi491538798 sequence were designed to amplify the full length Amy63 encoding sequence. The amplified DNA was ligated to the pGEM-T vector (Tiangen, China) for sequencing.

For sequence analysis of Amy63, sequences of ten typical α-amylases from GH13 subfamilies 1, 5, 6, 7, 15, 24, 27, 28, 36, 37 together with Amy63 were aligned using the program Clustal-W2^37^. Sequence logos of CSRs of α-amylases created using the WebLogo 3.0 server^38^.

### Cloning, expression and purification of Amy63 in ***E. coli***.

The full-length *amy63* DNA fragment with artificial *Eco*RI and *Hin*dIII sites was amplified by PCR using primers 63F and 63R. The PCR products were gel-purified and cloned into pET-30a(+) vector. The positive recombinant was confirmed by sequencing and transformed into *E. coli* BL21 (DE3) cells, and induced with isopropyl-β-thiogalactopyranoside (IPTG) at a final concentration of 0.2 mM at 16 °C for 16 h. The recombinant Amy63 was purified as described before^39^. Briefly, the protein was purified with three-column step procedure: HisTrap^TM^ HP column (GE Healthcare), HiTrap^TM^ Q HP column (GE Healthcare) and Hiload^TM^ 16/600 superdex^TM^ 200 column (GE Healthcare). Further detailed purification was taken as that of native Amy63.

### Substrates Degradation analyses of Amy63

Amy63 (1 mg/ml) was incubated separately with 0.25% agar, 0.25% carrageenan or 1% soluble starch in 20 mM Tris buffer (pH 8.0) at 37 °C for 24 h and the product mixtures were measured by gel permeation chromatography (GPC). G-5000, G-3000 and KS-802 gel exclusion columns were used and eluted with 0.2 M NaNO_3_ at a flow rate of 0.6 ml/min. The columns in the GPC apparatus were thermostatted at 40 °C.

To determine the substrate specificity of amylase, the enzyme activity was assessed against 1% (w/v) of soluble starch, dextrin, amylose, amylopectin, α-cyclodextrin, β-cyclodextrin, glycogen and pullulan in acetic acid buffer (pH 6.0) at 50 °C using 3,5-dinitrosalicylic acid (DNS) method^40^. The quantitative determination of enzyme activity was measured by the release of the reducing sugar equivalent using DNS and the procedure was performed as follows: 1% soluble starch or other substrates were dissolved in 20 mM Tris buffer (pH 8.0), warmed at 50 °C, and mixed with Amy63 solution. After incubation at 50 °C for 30 min, DNS reagent was added followed by boiling the reaction for another 5 min. The reaction was cooled using flowing water. And the absorbance at 540 nm (OD_540_) was then recorded^39^. Enzyme activity (U) was defined as the amylase enzyme that liberated 1 μmol of D-glucose per minute.

For maltohexaose degradation assay, Amy63 (1 mg/ml) was incubated with 25 mM maltohexaose in 20 mM Tris buffer (pH 8.0) at 37 °C for 12 and 24 h, respectively, and the saccharide product mixtures were analyzed by hydrophilic interaction chromatography.

### Effects of pH and temperature on Amy63 amylase, agarase and carrageenase activities

The effect of pH on Amy63 amylase, agarase and carrageenase activities were performed at 50 °C in 0.2 M Glycine-HCl (pH 3.0, 4.0), sodium acetate buffer (pH 5.0, 6.0), Tris-HCl buffer (pH 7.0, 8.0), Glycine-NaOH buffer (pH 9.0, 10.0, 11.0) for 30 min with the method described by Suzuki *et al.*^15^. For the measurement of pH stability, the enzyme was pre-incubated at 4 °C for 12 h at pH 3.0, 4.0, 5.0, 6.0, 7.0, 8.0, 9.0, 10.0 and 11.0 in buffer solutions. The residual activity was measured under the standard DNS assay at optimum temperature for 30 min with the addition of 1% starch, 0.25% agar, 0.25% carrageenan for amylase, agarase and carrageenase activities assays, respectively.

To evaluate the optimal temperature of Amy63, the DNS assay which uses soluble starch, agar or carrageenan as substrates, respectively, was conducted at various temperatures ranging from 0 °C to 100 °C in acetic acid buffer (pH 6.0). The thermostability of Amy63 was determined by measuring the residual activity after pre-incubating them for 1 h at various temperatures ranging from 0 °C to 100 °C and the relative activity was defined as the percentage of activity against the highest activity^41^.

### Effects of metal ions, surfactant and chelating agent on Amy63 amylase, agarase and carrageenase activities

Effects of different concentrations (1 mM, 10 mM, 100 mM) of metal ions (KCl, NaCl, SrCl_2_, CaCl_2_, MgCl_2_, MnCl_2_, FeSO_4_), surfactant (SDS) and chelating agent (EDTA) on Amy63 amylase, agarase and carrageenase activities were evaluated by measuring the residual enzyme activity with DNS method described above. And the relative activity in the absence of any additives was taken as 100% as described by Mohammad *et al.*^42^.

### Physiological function assays of Amy63

In order to analyze the physiological function of Amy63, the *E. coli* BL21 (DE3) cells containing pET30a or pET30a/Amy63 (overexpress Amy63) were cultured in LB medium supplemented with 50 mg/l kanamycin until OD600 reached 0.8. Thereafter, the same amount of *E. coli* BL21 (DE3) cells containing pET30a or pET30a/Amy63 were taken out from the flasks and centrifuged in 6,000 rpm for 3 min, and the pellets were washed with basic medium (10 g/l peptone, 5 g/l NaCl, 1 g/l KNO_3_, 0.5 g/l K_2_HPO_4_, 0.5 g/l MgSO_4_·7H_2_O, and 0.01 g/l FeSO_4_·7H_2_O) three times. After the washes, the pellets were resuspended with the same amount of basic medium, and 1 ml of resuspension cells containing pET30a or pET30a/Amy63 was added into 50 ml basic medium supplemented with 50 mg/l kanamycin and 0.5% starch, 0.1% agar or 0.1% carrageenan, respectively. The cells were inoculated in 37 °C at 150 rpm. When the OD600 reached around 0.8, the cells were induced with IPTG at a final concentration of 0.2 mM and cultured at 25 °C with shaking for another 20 h. The OD600 values were checked every several hours after the IPTG was added. Each treatment was done with three independent replicates.

### Motif analysis and truncates construction, expression and purification

The motifs of Amy63 were analyzed by using the MOTIF Search program (http://www.genome.jp/tools/motif/). The UniProt accession numbers of α-amylases from different kingdoms of life used for GH70-1, GH70-2 and GH70-3 Weblogo analyses are as following: AGV20183.1, ACY52744.1, AEM05860.1, AIA26430.1, ACQ73554.1, AJH89352.1, AJH21062.1, AAB86961.1, BAA32431.1, AAW44866.1, YP_007313109.1, BAP33443.1, AIN23306.1, AGJ85226.1, AHB43819.1, AKF38621.1, AJJ12514.1, BAB71820.1, AKG37901.1, AKG73076.1, AEP00550.1.

Four truncates of Amy63 which lack the first GH70 homolog, the first two GH70 homologs, the third GH70 homolog or the DUF1939 motifs, respectively, were constructed using primers F1, F2, R2, R3, R4. After over-expression in *E. coli* BL21 (DE3) cells, those four mutants were purified using the procedures described above and used for further plate-based activity assays and zymogram analyses.

### Phylogenetic analysis of Amy63

The program MEGA version 6.0 was used to construct phylogenetic trees^43^ of Amy63 with other α-amylases. The motif searching for amylases was done by MOTIF Search program (http://www.genome.jp/tools/motif/).

## Additional Information

**How to cite this article**: Liu, G. *et al.* Amy63, a novel type of marine bacterial multifunctional enzyme possessing amylase, agarase and carrageenase activities. *Sci. Rep.* **6**, 18726; doi: 10.1038/srep18726 (2016).

## Supplementary Material

Supplementary Information
