# Supplementary material for: Amy63, a novel type of marine bacterial multifunctional enzyme possessing amylase, agarase and carrageenase activities
Source: Sci Rep. 2016 Jan 4;6:18726. doi: 10.1038/srep18726 (PMC4698717; doi:10.1038/srep18726)
Supplement: Supplementary Information [file srep18726-s1.pdf]

Amy63, a novel type of marine bacterial multifunctional enzyme possessing amylase, agarase and carrageenase activities

Ge Liu[1,2], Shimei Wu[3],Weihua Jin[1], Chaomin Sun[1,*]

[1]Key Laboratory of Experimental Marine Biology, Institute of Oceanology, Chinese Academy of Sciences, Qingdao 266071, China

[2] The University of Chinese Academy of Sciences, Beijing, 100049, China.

[3]Qingdao Institute of Bioenergy and Bioprocess Technology, Chinese Academy of Sciences, Qingdao 266101, China

[*]Corresponding author

Chaomin Sun Tel.: +86 532 82898857; fax: +86 532 82898648.

E-mail addresses: sunchaomin@qdio.ac.cn

Supplementary information

a

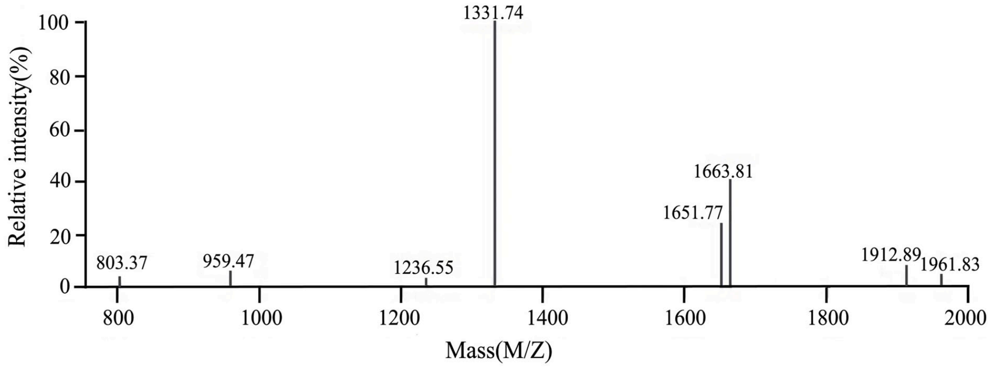

b Amy63

1 MKTFNLKPTL LPLTLLLSSP VMAAQNGTMM QYFHWYVPND GALWTQVENN
51 ASALSDNGFT ALWLPPAYKG AGGSNDVGYG VYDMYDLGEF DQQGSVRTKY
101 GTKDQYLSAI NTAHKNNIQI YGDVVFNHRG GADGKSWVDT KRVDWNNRNI
151 ELGDKWIEAW VEFDFPGRND KYSNFHWTWY HFDGVDWDDA GEEKAIFKFK
201GEGKAWDWEV SSEKGNYDYL MYADLDMDHP EVKQELKDWG EWYINMTGVD
251GFRMDAVKHI KYQYLQEWID HLRWKTGKEL FTVGEYWNYD VNQLHNFITK
301TSGSMSLFDA PLHMNFYNAS KSGGSYDMRQ IMDGTLMKDN SVKAVTLVEN
351HDTQPLQALE STVDWWFKPL AYAFILLREE GYPSVFYADY YGAQYSDKGH
401DINMVKVPYI EELVTLRKDY AYGKQHSYLD HWDVIGWTRE GDAKHPHSMA
451VIMSDGPGGS KWMYTGKPSA RYVDKLGIRT EEVWTDANGW AEFPVNGGSV
501SVWVSVE

c

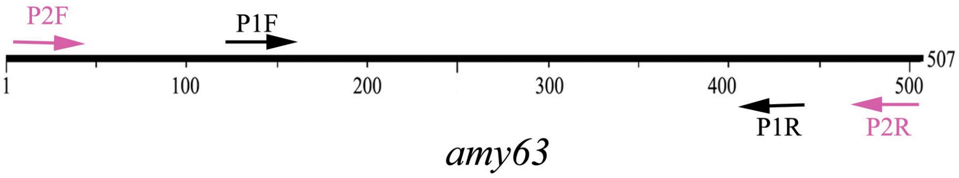

Figure S1. MALDI-TOF/TOF analysis of Amy63 and gene cloning strategy of its encoding sequence. (a) Fragment mass spectrum of Amy63. (b) Mass spectrum sequence coverage of Amy63. The peptides identified in the mass spectrum are indicated in red. (c) Gene cloning strategy of *amy63*. The sequences of P1F, P1R, P2F and P2R are shown in the Supplemental Table S1.

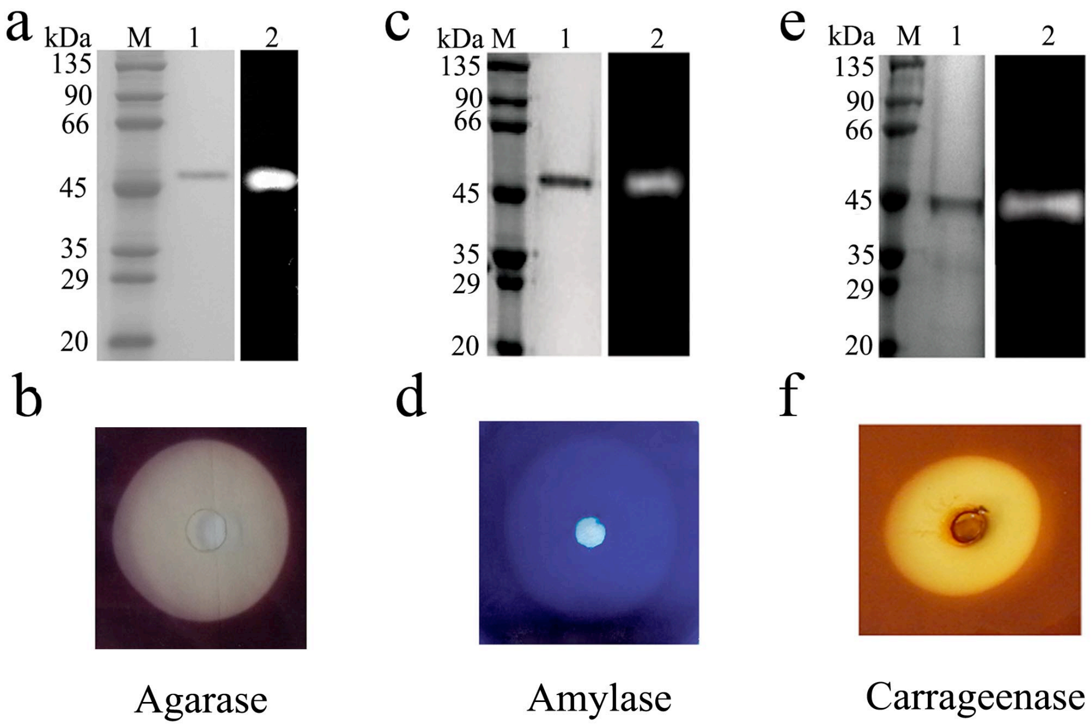

Figure S2. Zymogram analyses and plate-based activity assays of agarase, amylase and carrageenase activities of recombinant Amy63. Zymogram analyses for agarase (a), amylase (c) and carrageenase (e) activities of recombinant Amy63; Plate-based activity assays of agarase (b), amylase (d) and carrageenase (f) of recombinant Amy63. Lane M, molecular mass markers; Lane 1, purified recombinant Amy63; Lane 2, zymogram of purified recombinant Amy63.

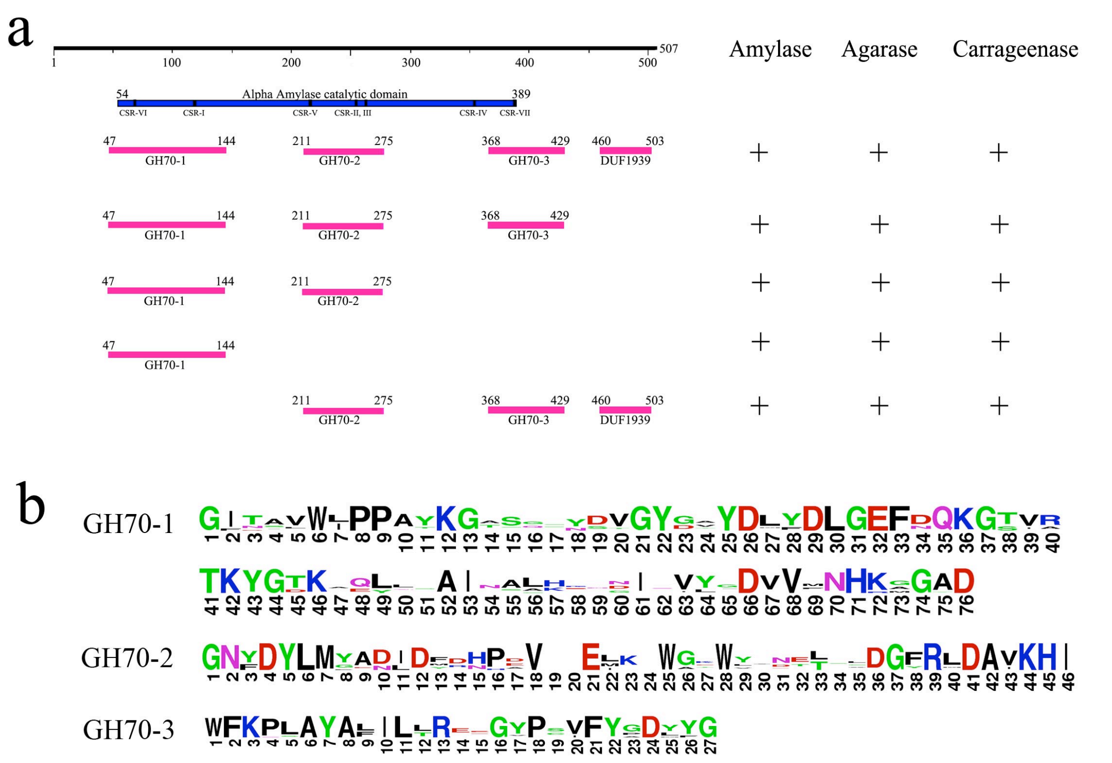

Figure S3. Function and sequence analyses of three GH70 homologs of Amy63. (a) Multifunction domain determination of Amy63. The truncations and their having corresponding enzyme activities are indicated with an +. The numbers represent the amino acid numbers of the corresponding protein and domains. (b) Sequence logos of three GH70 homologs based on the mixture of Amy63 and various α-amylases. The UniProt accession numbers of α-amylases from different kingdoms of life for GH70-1, GH70-2 and GH70-3 WebLogo analyses were shown in the Methods. The size of the single letter amino acid code in each sequence logo represents the occurrence of a particular amino acid at a particular position.

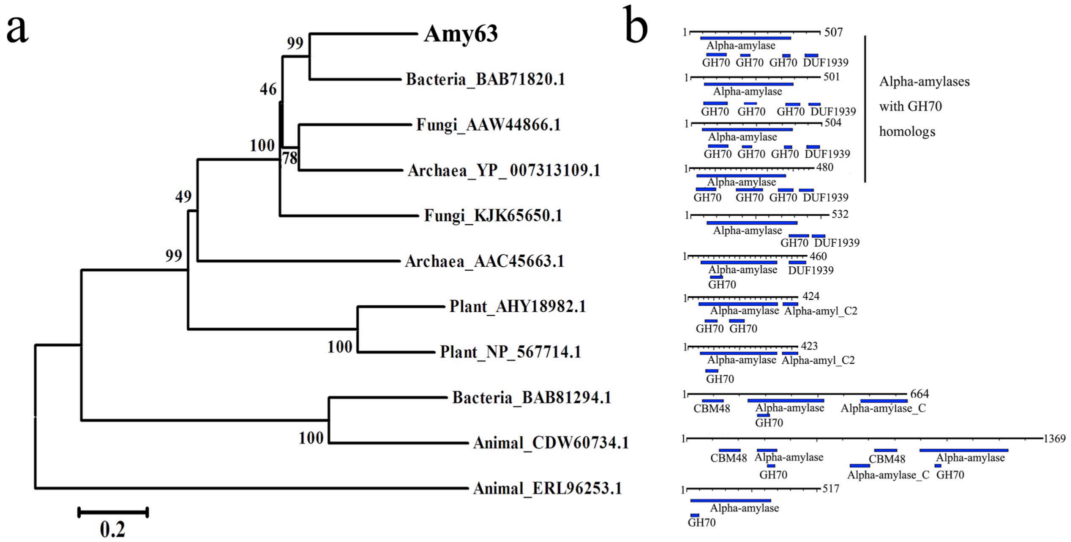

Figure S4. Phylogenetic analysis of Amy63 with GH70 homologs containing α-amylases from three different kingdoms of life. (a) Unrooted phylogenetic tree of Amy63 with GH70 homologs containing α-amylases from three different kingdoms of life. The UniProt accession numbers of α-amylases used for the phylogenetic tree are shown in the Methods. Alignments and dendrogram construction were carried out with MEGA version 6.0, using the neighbor joining method. Bootstrap values are given at the branching points. The bar corresponds to a genetic distance of 0.2 substitution per position (20% amino acid sequence difference). The UniProt accession numbers of α-amylases from different kingdoms of life are as following: AAW44866.1 (fungi), YP_007313109.1 (archaea), KJK65650.1 (fungi), BAB71820.1 (bacteria), BAB81294.1 (bacteria), AAC45663.1 (archaea), AHY18982. (plant), NP_567714.1 (plant), CDW60734.1 (animal), ERL96253.1 (animal). (b) The corresponding domain organization of α-amylases used in panel a. There is a one-to-one relationship between panels a and b. All the domain organizations of GH13 subfamily α-amylases were analyzed with MOTIF Search program.

Supplementary Table 1. Primers used in this study.

Primer name	Primer sequence (5’- 3’)
27F	AGAGTTTGATCCTGGCTCAG
1541R	AAGGAGGTGATCCACCC
P1F	CGVGT(M)GATTGGAACAACCGM
P1R	RTGGTCGAGATAAGAATGTTG
P2F	ATGAAAACATTCAACCTTAAACC
P2R	TTAYTCAACGCYAACCCAAMM
63F	CGGAATTCATGAAAACATTCAACC
63R	CCAAGCTTTTCAACGCTAACCCAA
F1	CGGAATTCATGAAAACATTCAACC
F2	CGGA ATTCTGGAACAACCGAAAT
R2	CCAAGCTTGCCGCCAGGTCCATCA
R3	CCAAGCTTAAACC ACCAATCAACC
R4	CCAAGCTTCACTTCCCAATCCCAT
